# A novel mutation in the *FGG* gene causes hypofibrinogenemia in a Chinese family

**DOI:** 10.1186/s41065-024-00313-3

**Published:** 2024-02-20

**Authors:** Xiaoying Xie, Juan Du, Shunkang Geng, Baoqin Yi, Qingpu Li, Jiangcheng Zuo

**Affiliations:** Department of Clinical Laboratory, Yichang Yiling People’s Hospital, Yichang, Hubei 443100 China

**Keywords:** FGG, Hypofibrinogenemia, Whole-exome sequencing

## Abstract

Congenital fibrinogen disorders are a group of coagulation deficiencies caused by fibrinogen defects and are divided into four types, including afibrinogenemia, hypofibrinogenemia, dysfibrinogenemia, and hypodysfibrinogenemia. In this study, we collected a family with hypofibrinogenemia, and genetics analysis identify a novel pathogenic variants (c.668G > C, p.Arg223Thr) in the *FGG* gene. And electron microscope observation revealed significant changes in the ultrastructure of fibrin of the proband. Our research expands the phenotypic and genetic spectrum associated with the *FGG* gene, which would facilitate in genetic counselling and prenatal genetic diagnosis.

**Supplementary Information:**

The online version contains supplementary material available at 10.1186/s41065-024-00313-3.

## Introduction

Fibrinogen is a hexamer composed of three proteins, (AαBβγ)_2_, and plays an essential role accompanied with its derivative fibrin in various biological processes such as cellular and matrix interactions, angiogenesis, blood clotting, wound healing, inflammation, and even in neoplasia [1, 2]. The genes encoding Aα, Bβ, and γ are *FGA*, *FGB*, and *FGG* respectively. Mutations in these genes can lead to congenital fibrinogen disorders (CFDs), which are inherited in an autosomal recessive or autosomal dominant manner. The clinical manifestations of CFDs exhibit significant variability, with some patients exhibiting bleeding and/or thrombosis, while others may be asymptomatic [3].

Different detection platforms have different reference values, but the normal levels of fibrinogen in the blood typically range between 1.5 and 4.3 g/L [3–5]. CFDs can divide into two groups based on deficiencies and/or defects in the fibrinogen molecule. Type I, recognized as quantitative disorders, are distinguished by decreased fibrinogen levels, include afibrinogenemia (undetectable or nearly undetectable fibrinogen levels, <0.1 g/L) and hypofibrinogenemia (reduced fibrinogen levels ranging from 0.1 to 1.5 g/L). Type II, recognized as qualitative disorders, include dysfibrinogenemia (normal fibrinogen levels with diminished functional activity) and hypodysfibrinogenemia (decreased fibrinogen levels coupled with diminished functional activity) [3–6]. And Fg:C/Fg:Ag ratio < 0.7 is considered one of the primary indicators for the diagnosis of dysfibrinogenemia, as a ratio > 0.7 indicates that the diminished functional activity of fibrinogen is attributed to the reduced fibrinogen levels rather than compromised quality [7]. Detection of CFDs can be challenging due to the significant variability of the clinical manifestations, with some patients exhibiting bleeding and/or thrombosis, while others may be asymptomatic [8].

In this study, we collected a three-generation family consisting of ten members and four members suffered from hypofibrinogenemia. Genetics analysis revealed that all patients in this family carried a heterozygous variant (c.668G > C, p.Arg223Thr) in the *FGG* gene. Structural analysis indicated that this variant may impact the stability of the FGG protein. Fibrin of the proband received electron microscope observation and revealed significant changes in the ultrastructure of the fibrin clots. Our finding expands our understanding of the phenotype-genotype correlations associated with the *FGG* gene.

## Materials and methods

### Coagulation testing and thromboelastography

Venous blood samples were collected form family members. Further investigations of coagulation function were performed. Prothrombin time (PT), thrombin time (TT), activated partial thromboplastin time (APTT), PT-derived assay and fibrinogen Clauss assay (Fg:C) were measured using automated coagulation analyzers (TOP550, Werfen, Spain) with ready-to-use hemostasis assays liquid RecombiPlasTin (0020002900, Werfen, Spain), HemosIL APTT SP (0020006300, Werfen, Spain), Thrombin Time (0009758515, Werfen, Spain), and Fibrinogen-C assays kit (0008469110, Werfen, Spain), separately. The D-Dimer was measured using an automatic fluorescent immunoassay system (Vidas, Biomerieux, France) with VIDAS D-Dimer Exclusion II assays kit (REF30455, Biomerieux, France). The antigen levels of fibrinogen (Fg:Ag) were measured using an special protein analyzer (BNProSpec, Siemens, Germany) by immunoturbidimetric assays method with Fibrinogen Assay Kit (OSCA09, Siemens, Germany). Thromboelastography assays were performed using kaolin and CaCl_2_ on the hemostasis system (CFMS LEPU-8800, Lepumedical, China).

### Genetics analysis

Genomic DNA were extracted from peripheral blood samples obtained from family members. Genomic DNA of the proband (III:1) underwent processing using the xGEN Exome Research Panelv 1.0 capture kit (Integrated DNA Technologies, USA) and the TruePrep Flexible DNA Library Prep Kit for Illumina (Vazyme, China). Subsequently, whole-exome sequencing was performed on an Illumina NovaSeq 6000 sequencer (Illumina, USA). High-quality reads were aligned to the human reference genome (hg19), excluding intergenic, intronic, and UTR region variations, as well as synonymous mutations and variations with a minor allele frequency (MAF) score exceeding 0.5%. For Sanger sequencing, forward primer (5′-TGGCTGGATGTGCTGTTTGC-3′) and reverse primer (5′-TGGCCAAGATCACTTAGTTGGGA-3′) were used for amplifying exon 7 of the *FGG* gene.

### Structure prediction

The FGG protein sequence was obtained from the Ensembl website (https://ensembl.org/). The domains of the FGG protein were predicted using Pfam (http://pfam.xfam.org/). The wild-type protein structures were predicted using AlphaFold2 (v2.2.3), provided by the Shenzhen Bkunyun Cloud computing platform (https://www.bkunyun.com/). The results of predicted protein structure were analyzed by PyMol (v2.5).

### Scanning electron microscopy observations

Fifty microliters (50 µl) of plasma samples from the proband and a healthy control were collected. Thrombin was added to the samples, resulting in a final concentration of 2 U/ml, and the mixtures were then incubated at 37 °C for 3 h. Fibrin clots were carefully selected and subjected to observation of their ultrastructure using a scanning electron microscope (Regulus8100, Hitachi, Japan). The diameter of the fibrin fibers was measured using Image-Pro Plus 6.0, and the data were analyzed using Prism GraphPad 8.0.

### Case report

**Fig. 1:**
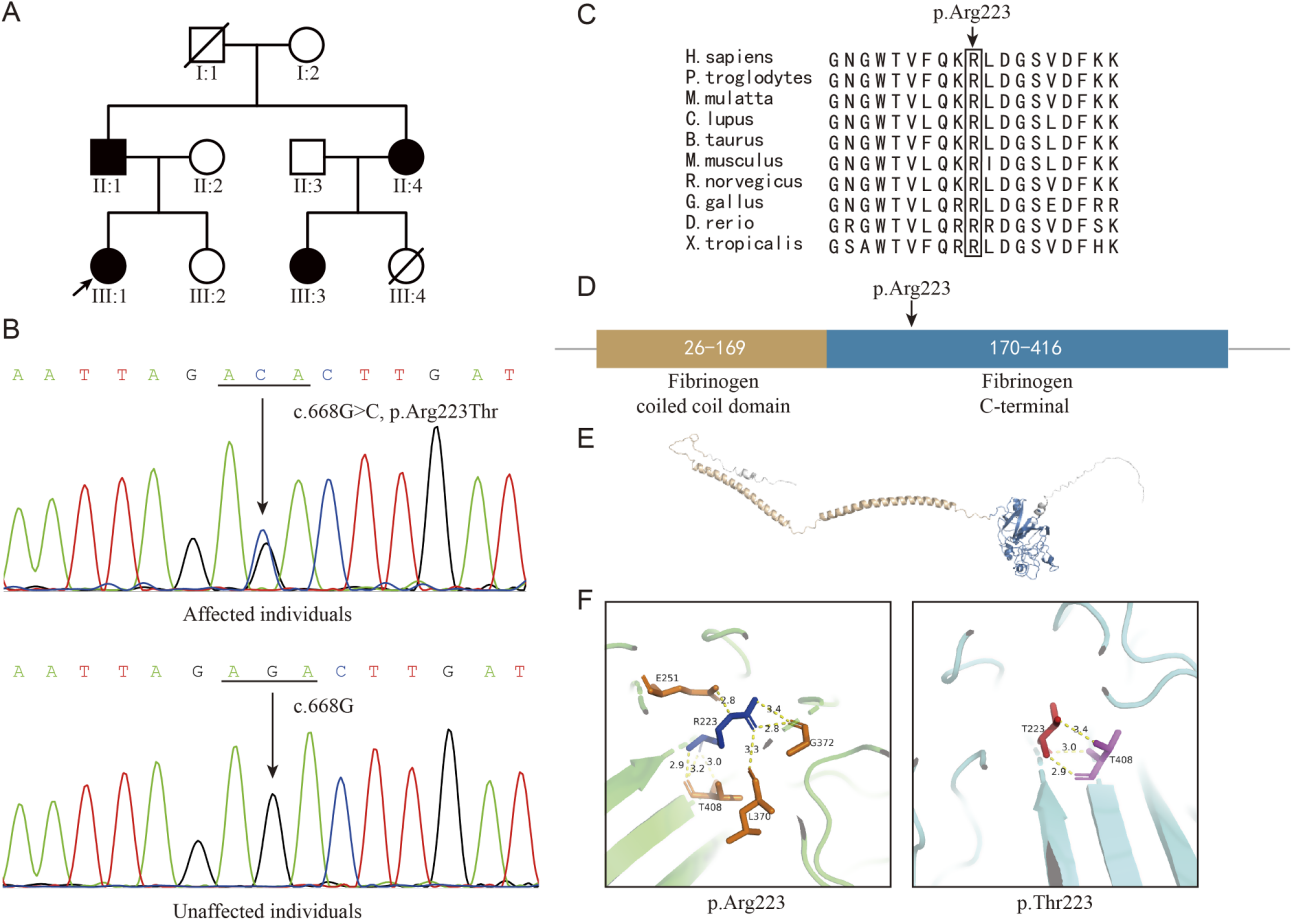
Variant in the *FGG* gene of the hypofibrinogenemia family and its effects on the protein structure and fiber. **A** Filled symbols represent affected individuals, while empty symbols represent unaffected individuals. The proband (III:1) is indicated by an arrow. **B** Sanger sequencing of affected and unaffected individuals. All patients carried the heterozygous mutation (c.668G > C, p.Arg223Thr) in the *FGG* gene. **C** Amino acid sequence alignment of FGG protein revealed that the p.Arg223 is highly conserved across different species. **D** Predicted protein domains of wild-type FGG. The fibrinogen coiled coil domain is depicted in wheat, while the fibrinogen C-terminal domain is depicted in sky blue. The p.Arg223 residue locates in the fibrinogen C-terminal domain. **E** The predicted protein structure of wild-type FGG, with the coiled-coil domain shown in wheat and the C-terminal domain shown in sky blue. **F** Hydrogen bonds are represented by yellow dotted lines. The p.Arg223 residue is displayed in blue, with residues forming hydrogen bonds shown in orange. The p.Thr223 residue is displayed in red, with residue forming hydrogen bonds shown in magenta

A three-generation family consisting of 10 members with four members suffered from hypofibrinogenemia was recruited (Fig. 1A). The proband (III:1), a 28-year-old female, was identified during a physical examination due to a prolonged bleeding time after blood collection. Self-reported experience of the proband including occasional bruising of the skin and mucous membranes, as well as a slightly prolonged bleeding following traumatic events. The proband had no history of spontaneous bleeding from joints or soft tissues, such as hematoma, nosebleeds, gum bleeding, or gastrointestinal bleeding; nor did she had menstrual disorders, prolonged menstrual periods, or bleeding or thromboembolism related to surgical procedures. However, the proband had normal blood test results, liver function, and kidney function. Liver function and blood tests of other family members were also normal, suggesting normal hepatocyte protein production and no infection were present in these individuals.

Individuals II:1, II:4, III:1 and III:3 exhibited prolonged TT (18.6 s, 18.8s, 19.4 and 22.5 s, separately); these four patients also exhibited reduced Fg:C (1.3 g/L, 1.45 g/L, 0.72 g/L and 0.85 g/L, separately), decreased levels of Fg:Ag (1.35 g/L, 1.51 g/L, 0.779 g/L and 0.769 g/L, separately), and decreased PT-derived method results (1.51 g/L, 1.67 g/L, 0.8 g/L and 0.97 g/L, separately). All patients are diagnosed with hypofibrinogenemia as their Fg:C/Fg:Ag > 0.7. Interestingly, when comparing the impact on fibrinogen between affected individuals of the third-generation and the second-generation, the Fg:C and Fg:Ag levels merely amount to half in the former. Detailed results of coagulation testing, thromboelastography results, blood test results and liver function results of this family are presented in Table 1.

**Table 1:** Coagulation testing results, thromboelastography results, blood test results and liver function results

	Test	I:2	II:1	II:2	II:4	III:1	III:2	III:3	Reference interval
Coagulation testing results	PT	11.8	12.9 ↑	11.3	11.4	12.6 ↑	11.8	14.9 ↑	9.4–12.5s
	APTT	27.7	33.9	32.1	29.5	39.2 ↑	36.5	30.8	25.1–36.5s
	TT	15.3	18.6 ↑	15.3	18.8 ↑	19.4 ↑	13	22.5 ↑	10.3–16.6s
	PT-derived	4.65	1.51 ↓	3.61	1.67 ↓	0.8 ↓	/	0.97 ↓	2.38–4.98 g/L
	Fg:C	3.3	1.3 ↓	2.96	1.45 ↓	0.72 ↓	2.57	0.85 ↓	2.38–4.98 g/L
	Fg:Ag	2.93	1.35 ↓	3.59 ↑	1.51 ↓	0.779 ↓	2.53	0.769 ↓	1.8–3.5 g/L
	D-Dimer	0.65 ↑	0.13	0.17	0.22	0.1	0.17	0.05	0–0.5 µg/mL
Thromboelast-ography results	R	/	1.8	/	6.1	7.9	5.8	2.8 ↓	4–9 min
	K	/	8.4 ↑	/	8.2 ↑	10.9 ↑	2.4	13.7 ↑	1–3 min
	Angle	/	33.8 ↓	/	30.2 ↓	25.1 ↓	56	26.4 ↓	53–72 deg
	MA	/	35.2 ↓	/	37 ↓	27.8 ↓	54	27.3 ↓	50–70 mm
Inflammatory markers	WBC	4.65	9.25	5.69	6.38	6.06	4.54	8.72	3.5–9.5 g/L
	CRP	1.04	5.92	2.38	1.14	1.6	1.17	2.31	0–10 mg/L
	SAA	< 4.80	5.38	< 4.80	< 4.80	< 4.80	5.56	< 4.80	0–10 mg/L
	PCT	< 0.05	< 0.05	< 0.05	< 0.05	< 0.05	< 0.05	< 0.05	0–0.05 mg/mL
Liver function	AST	21	34	30	21	19	14	18	13–35 U/L
	ALT	9	34	30	12	21	13	16	7–40 U/L
	TP	73.6	69	73.6	71.9	75	68.8	70.8	65–85 g/L
	ALB	45.3	45.8	44.1	46.2	51	46	46.5	35–55 g/L

*Abbreviations* PT, prothrombin time; APTT, activated partial thromboplastin time; TT, thrombin time; Fg:C, activity of fibrinogen measured by Clauss assay; Fg:Ag, antigen level of fibrinogen; WBC, white blood cell; CRP, C-reactive protein; SAA, serum amyloid A; PCT, procalcitonin; AST, aspartate aminotransferase; ALT, alanine aminotransferase; TP, total protein; ALB, albumin. ↑, above; ↓, below

A novel variant (c.668G > C, p.Arg223Thr) was identified in the *FGG* gene by whole-exome sequencing and was further validated by Sanger sequencing. All affected individuals in this family carried the heterozygous mutation (c.668G > C, p.Arg223Thr) in the *FGG* gene, whereas unaffected individuals did not (Fig. 1B). The p.Arg223 residue is conserved across different species (https://www.ncbi.nlm.nih.gov/homologene/) (Fig. 1C), which implied that it had an important function. The domains of wild-type FGG was predicted, as the fibrinogen coiled coil domain is depicted in wheat, while the fibrinogen C-terminal domain is depicted in sky blue; and the p.Arg223 residue locates in the fibrinogen C-terminal domain (Fig. 1D). The protein structure of FGG was also predicted, as the coiled-coil domain of fibrinogen is represented in wheat, while the fibrinogen C-terminal domain is represented in sky blue (Fig. 1E); the results revealing that the p.Arg223 residue could form eight hydrogen bonds with four surrounding residues, whereas p.Thr223 could only form three bonds with one residue (Fig. 1F). This difference in hydrogen bonds might impact the stability of the FGG protein and further affect the level of fibrin. Furthermore, since the variant is located in the fibrinogen C-terminal domain involved in fibrin assembly [9], the p.Arg223Thr mutation may also impact the levels of fibrin by affecting protein assembly.

**Fig. 2:**
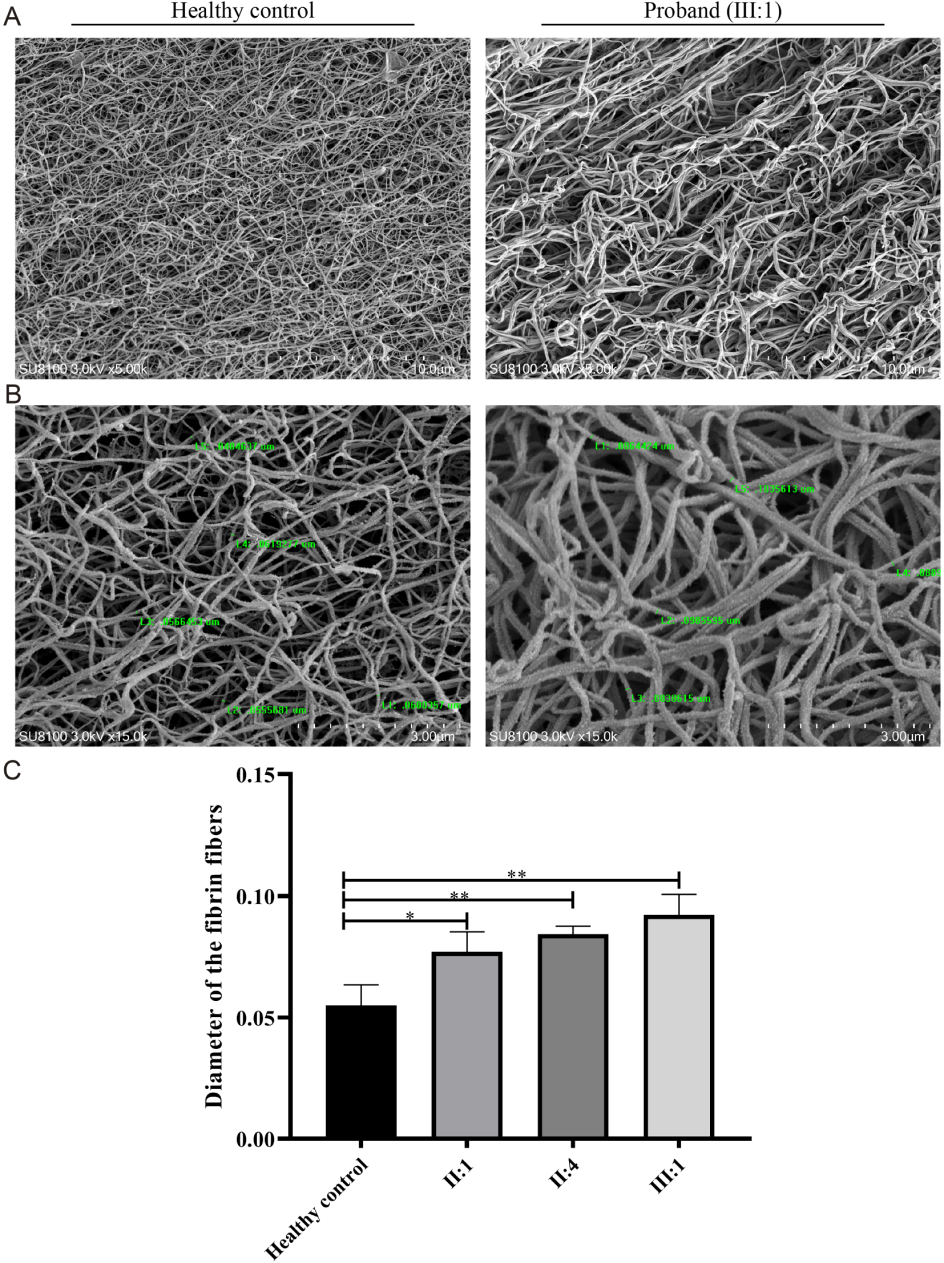
Scanning electron microscopy of fibrin clots. **A** Ultrastructure of the fiber network space from the proband (left) and healthy control (right). **B** Measurement of fiber filament diameter and their comparison between the proband (left) and healthy control (right). **C** Comparation of the diameter of the fiber filaments. “*” represents *p* < 0.05 and “**” represents *p* < 0.01

Fibrin clots formed using plasma samples of the proband and a healthy control were observed using a scanning electron microscope. Compared to the healthy control, the proband exhibited a loose fiber network spacing structure with a significantly larger central hole (Fig. 2A). The fibrinogen from patient II:1 and II:4 were also observed by scanning electron microscopy; the density of the fiber network of these two patients were lower than the healthy control but were higher than the proband (Fig. S1A). Additionally, the diameter of the fiber filaments was thicker, measuring 91.49 ± 8.05 nm, compared to 61.22 ± 9.322 nm in the healthy control (Fig. 2B). Moreover, an assessment of fiber filament diameter revealed that all patients exhibited thicker fiber filaments than the healthy control (Fig. 2C), while there was no significant difference among the three patients (Fig. S1B). The causes of fibrin abnormalities including impaired release of fibrin peptide A/B, impaired polymerization of fibrin monomers, or impaired XIIIa-mediated cross-linking [10]. Scanning electron microscopy observations of the fibrin from the proband suggested that the strength, structure, and stability of fibrin might be impaired. Consequently, the clot formed by such fibrin is more permeable and less likely to effectively entangle platelets and other blood cells, resulting in reduced hemostasis and an elevated risk of bleeding compared to healthy individuals [11].

## Discussion

Patients diagnosed with CFDs commonly exhibit reduced levels of fibrinogen quantity and/or quality, along with decreased fibrinogen activity levels. Patients II:1, II:4, III:1 and III:3 exhibited reduced Fg:C (1.3 g/L, 1.45 g/L, 0.72 g/L and 0.85 g/L, separately) and decreased levels of Fg:Ag. (1.3 g/L, 1.45 g/L, 0.72 g/L and 0.85 g/L, separately). The levels of fibrinogen were also assessed using the PT-derived method, an indirect approach that determines fibrinogen levels by calculating changes in plasma turbidity [12], also showed decreased in these four patients (1.51 g/L, 1.67 g/L, 0.8 g/L and 0.97 g/L, separately).

For quantitative fibrinogen disorders, individuals with Fg:C > 1.0 g/L typically remain asymptomatic, while Fg:C < 0.7 g/L can lead to spontaneous or traumatic bleeding; and for qualitative fibrinogen disorders [4]. In this study, patients II:1 and II:4, with Fg:C > 1.0 g/L, only presented with mildly prolonged TT. Patient II:4 underwent two caesarean sections without bleeding or thrombosis. However, patients III:1 and III:3, with Fg:C < 1.0 g/L, exhibited prolonged TT and PT. Although patients III:1 and III:3 did not show signs of spontaneous bleeding, they had significantly longer bleeding following traumatic events.

Based on the clinical presentations and coagulation test results, the severity of fibrinogen abnormalities and clinical phenotypes in this family appears to increase across generations. In fact, there is a strong correlation between the severity of impaired FGG protein function and the severity of CFDs. Arbez et al. reported a patient carrying a homozygous mutation in the *FGG* gene, p.Arg223Term, in the same position as observed in this study. The nonsense mutation caused an early termination, potentially disrupting the hexameric fibrinogen. Consequently, this patient exhibited a more severe clinical phenotype of afibrinogenemia due to the absence of fibrinogen [13].

Thromboelastography of the affected individuals within this family revealed increased K values and decreased Angle values and MA values, suggesting decreased fibrinogen activity and defective fibrin function [14]. The clot strength relies on platelets and fibrinogen, and platelets bind to fibrin through glycoprotein IIb/IIIa and contribute to clot contraction through cytoplasmic motor proteins within the platelets [15]. In this family, the number and function of platelets were normal, but the MA values were lower than the reference interval. This suggests that abnormal fibrinogen may have reduced capacity to bind to activated platelets, ultimately leading to insufficient clot strength.

In conclusion, our study identified a novel mutation (c.668G > C, p.Arg223Thr) in the *FGG* gene that causes hypofibrinogenemia, expanding the genetic spectrum and contributing to prenatal diagnosis of congenital fibrinogen disorders. And we found it interesting that the reduction in fibrinogen levels and the appearance of clinical phenotypes were more severe in the third-generation compared with the second-generation. Although fibrinogen levels could be influenced by many variables such as fibrinogen polymorphisms, age, and sex; and many large families with CFDs have been reported, none with an increase in severity over generations. However, whether the observed pattern in our study is a coincidence or a generalized pattern due to an underlying cause that we may have overlooked, needs to be further investigated by enriching more cases.

### Electronic supplementary material

Below is the link to the electronic supplementary material.

Supplementary Material 1

## Data Availability

This work did not generate new datasets.

## Abbreviations

CFDs: congenital fibrinogen disorders
PT: Prothrombin time
TT: thrombin time
APTT: activated partial thromboplastin time
Fg: C:fibrinogen Clauss assay
Fg: Ag:antigen levels of fibrinogen
