# Supplementary figures and images for: A novel mutation in the FGG gene causes hypofibrinogenemia in a Chinese family
Source: Hereditas. 2024 Feb 20;161:9. doi: 10.1186/s41065-024-00313-3 (PMC10877905; doi:10.1186/s41065-024-00313-3)

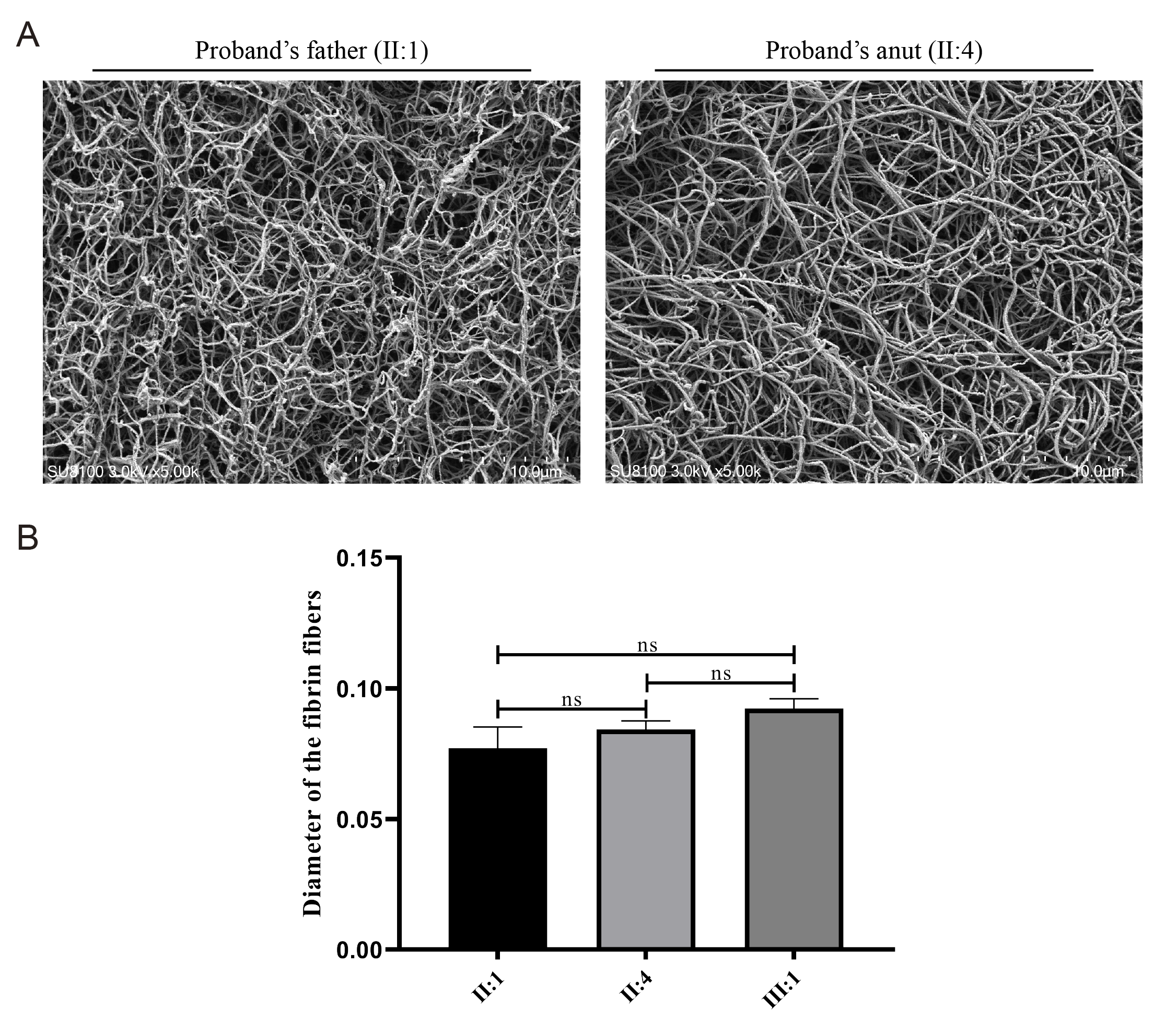

Supplement: Supplementary file 1 — Supplementary Material 1 [file 41065_2024_313_MOESM1_ESM.png]
